# A Rare Chromosome 3 Imbalance and Its Clinical Implications

**DOI:** 10.1155/2012/846564

**Published:** 2012-10-11

**Authors:** Karen Sims, Roberto L. P. Mazzaschi, Emilie Payne, Ian Hayes, Donald R. Love, Alice M. George

**Affiliations:** ^1^Diagnostic Genetics, LabPlus, Auckland City Hospital, P.O. Box 110031, Auckland 1148, New Zealand; ^2^Genetic Health Service of New Zealand-Northern Hub, Auckland City Hospital, Private Bag 92024, Auckland 1142, New Zealand; ^3^School of Biological Sciences, University of Auckland, Private Bag 92019, Auckland 1142, New Zealand

## Abstract

The duplication of chromosome 3q is a rare disorder with varying chromosomal breakpoints and consequently symptoms. Even rarer is the unbalanced outcome from a parental inv(3) resulting in duplicated 3q and a deletion of 3p. Molecular karyotyping should aid in precisely determining the length and breakpoints of the 3q+/3p− so as to better understand a child's future development and needs. We report a case of an infant male with a 57.5 Mb duplication from 3q23-qter. This patient also has an accompanying 1.7 Mb deletion of 3p26.3. The duplicated segment in this patient encompasses the known critical region of 3q26.3-q27, which is implicated in the previously reported 3q dup syndrome; however, the accompanying 3p26.3 deletion is smaller than the previously reported cases. The clinical phenotype of this patient relates to previously reported cases of 3q+ that may suggest that the accompanying 1.7 Mb heterozygous deletion is not clinically relevant. Taken together, our data has refined the location and extent of the chromosome 3 imbalance, which will aid in better understanding the molecular underpinning of the 3q syndrome.

## 1. Introduction

The genetic analysis of infants with multiple congenital abnormalities is a very important aid in understanding a child's future prognosis and development. Microarray technologies are more commonly becoming the tool of choice to accurately determine the underlying genetic cause and resulting phenotype [1].

The duplication of chromosome 3q is a rare genetic disorder resulting in mental retardation, seizures, broad nose, cardiac, renal, and genital malformations [2]. The critical region of 3q+ has been defined by Aqua et al. [3] as 3q26.31-q27.3. In contrast, deletions of chromosome 3p are associated with intrauterine and postnatal growth retardation with delayed bone maturation, severe psychomotor retardation, dysmorphism including ptosis, a narrow nose, flat nasal bridge, clinodactyly, heart and kidney defects, and impaired vision [2, 4]. The size of the deletion appears to correlate with severity of the phenotype such that patients with a large deletion exhibit severe malformations and mental retardation [5]. The reported breakpoints for the 3p− syndrome appear to be variable, but the 3p− phenotype is associated with deletions in the 3pter-3p25 region [6]. 

Previously reported cases of patients carrying a duplication at 3q23-ter as well as a large deletion at pter-3p25 have a fatal outcome [2]. We report a case here in which the patient has a much smaller 3p deletion in combination with the 3q23-ter duplication, and discuss whether the 3p deletion size affects patient phenotype and outcome.

## 2. Case Report

A one-month-old male presented with a large ventricular septal defect (VSD), large posterior and anterior fontanelle, dysmorphic features, single palmar crease, under-developed testes and mild seizures. The baby was the product of a normal first pregnancy and was delivered at 41 weeks, 6 days with a birth weight of 4.1 kgs (9 lbs). Labour was complicated by foetal distress, and delivery was by caesarean section and admitted to the Newborn Intensive Care Unit (NICU) on day 2 for respiratory distress.

Ultrasound analysis revealed a thin corpus callosum and a consequent MRI of the brain and spine revealed a small right germinal matrix haemorrhage and mild craniofacial disproportion and mild micrognathia. There was also a separate choroidal fissure cyst on the left 9 mm in maximal dimension. The corpus callosum was thin, but present.

At 14 months of age, the proband had gained weight despite feeding difficultes and was breast feeding; post surgery, the child exhibited normal biventricular function with no residual VSD and no audible murmurs; he was tachypnoeic with a respiratory rate of 60, but his chest was clear. He had a thickened filum, and surgery was suggested in infancy to prevent later problems with foot development. He also had reduced antigravity movement in his upper and lower limbs with reduced central tone, but increased tone in his limbs.

### 2.1. Cytogenetic and Microarray Analysis

**Figure 1:**
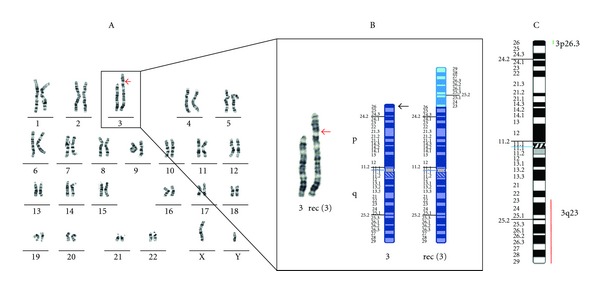
Karyotype and chromosome 3 ideogram of the proband. Panel A shows the karyotype of the proband, 46,XY,rec(3)dup(3q)inv(3)(p26.3q23)mat.arr 3p26.3(56,669-1,850,707)x1,3q23q29(141,829,104-199,355,203)x3. Panel B shows the normal and derivative chromosomes 3, together with an associated ideogram. Panel C is a summary ideogram of the regions of chromosome 3 that are duplicated and deleted in the proband.

Metaphase chromosomes were prepared from stimulated peripheral blood cells according to standard methods and karyotyping was performed by G-band metaphase analysis. This analysis showed a large duplication of material on the short arm of chromosome 3, which appeared 3q-like (Figure 1).

**Figure 2:**
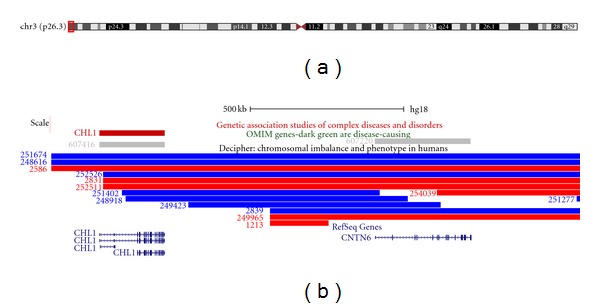
Schematic of the deleted chromosome 3 region in the proband. Panel A shows an ideogram of chromosome 3, together with the location of the deletion indicated in red. Panel B shows the genes that are localised within the deleted region, those reported in the OMIM database (http://www.ncbi.nlm.nih.gov/omim/) together with the location and extent of duplications (shown in blue) and deletions (shown in red) in patients reported in the DECIPHER database (http://decipher.sanger.ac.uk/). The images presented here are taken from the UCSC genome browser (http://genome.ucsc.edu/).

Higher resolution molecular karyotype analysis was performed to determine the extent of the 3q+ region. Briefly, genomic DNA was isolated from peripheral blood using the Gentra Puregene blood kit according to the manufacturer's instructions (Qiagen Pty Ltd., MD, USA). 0.1 micrograms of genomic DNA was labelled using the Affymetrix Cytogenetics Reagent Kit and labelled DNA was applied to an Affymetrix Cytogenetics Array (2.7 million probes) according to the manufacturer's instructions (Affymetrix Inc, CA, USA). The array was scanned and the data analysed using the Affymetrix Chromosome Analysis Suite (ChAS; version 1.0.1) and interpreted with the aid of the UCSC genome browser (http://genome.ucsc.edu/; hg18 assembly). This analysis confirmed the copy number change as a 57.5 Mb terminal duplication from 3q23-qter, together with an unsuspected 1.7 Mb deletion at 3p26.3 (Figure 1). The deleted region contains two genes, *CNTN6* and *CHL1* (Figure 2).

**Figure 3:**
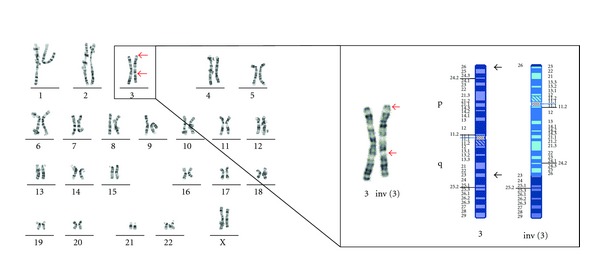
Karyotype and chromosome 3 ideogram of maternal chromosomes. Panel A shows the karyotype of the mother 46,XX,inv(3)(p26.3q23). Panel B shows the normal and structurally rearranged chromosomes 3, together with an associated ideogram.

Parental analysis confirmed that these chromosome 3 changes arose as an unbalanced product of a meiotic recombination in the mother who has a pericentric inversion of one homologue of chromosome 3 between p26 and q23 (Figure 3). 

Patients with 3p− who have been reported in the DECIPHER database exhibit a range of phenotypes (Table 1). The deletions in these patients range in length from 200 kb to 12.5 Mb, incorporating 42 known genes from 3pter-3p25. In the main, the associated clinical phenotypes of these patients do not match those identified in the proband who carries a smaller distal 3p deletion, as well as a duplication of 3q. The phenotypes that do match such as mental retardation/developmental delay, VSD, dysmorphism, and seizures are also symptoms that are associated with the 3q+ syndrome.

## 3. Discussion

The duplicated segment in the proband described here encompasses the known critical region of 3q26.3-q27 [3], which is implicated in the previously reported 3q+ syndrome; the proband exhibits the 3q+ syndrome phenotype [2, 3, 7]. The accompanying deleted region at 3pter is small and encompasses only two genes, *CNTN6* and *CHL1*. Both of these genes are implicated in psychomotor retardation [8, 9], which is a phenotype exhibited by patients with the 3q+ syndrome. 

Previously reported patients with a smaller 3p deletion at 3p26.1-3pter and 3p26.3-3pter exhibit a mild phenotype with no heart disease and mild or no mental retardation [9, 10], suggesting that these smaller deletions do not cause the 3p− phenotype. The 3p− phenotype has been well characterised and most reported cases have a larger deletion than the proband, from 3pter-p25 [4, 5, 11–13]. Cargile et al. [13] reported a patient with a small interstitial deletion at 3p25.3-p26.2 that had a 3p− clinical phenotype of ptosis, microcephaly, growth retardation, and developmental delay. Other reported cases of 3p− phenotypes are consistent with a larger deletion incorporating the 3p25.3-p26.2 region suggesting that the gene/genes contributing to the 3p− phenotype are within this region [9, 14–16]. Malmgren et al. (2007) reported that they consider this minimal region of overlap between the reported cases, which includes 12 genes, to contain the candidates for the 3p− phenotype. 

The many genes reported as potential candidate genes in the 3p− phenotype include *ATP2B2*, *CNTN4*, *ITPR1*, *LRRN1*,* SUMF1, *and *SRGAP3* [9, 16–18], which are present in the minimal region of overlap reported by Cargile et al. [13] at 3p25.3-p26.2; this region is not deleted in the proband described here. Evidence to suggest that genes in the 3p25.3-p26.2 region are involved in the 3p− phenotype is supported by a case with a *de novo* balanced translocation between chromosomes 3 and 10 that disrupted the *CNTN4* gene. This patient exhibited a 3p− phenotype [19].

Most patients reported with a deletion/duplication of chromosome 3 have a larger deletion from 3pter-p25 with a duplication at either 3p21 or 3p23 and have a very poor outcome with a clinical picture of growth deficit, delayed bone maturation, microcephaly, narrow nose and multiple malformations [2]. The clinical phenotype of our patient is more consistent with a diagnosis of the 3q+ syndrome phenotype alone. The patient had a high birth weight and broad nose, which is not consistent with the deletion phenotype. Microarray analysis confirmed that this patient has a smaller accompanying 1.7 Mb deletion from 3pter-p26.3. The size of the deletion appears to have minimal impact on the phenotype of this patient and this is consistent with previously reported cases. The principal conclusion, therefore, is that the patient's clinical progression should follow a 3q syndrome phenotype. 

## 4. Conclusion

Taken together, our data has refined the location and extent of chromosome 3 imbalances. Molecular karyotyping has led to a better understanding of the molecular underpinning and phenotypic outcome in the proband reported here and should be considered in future cases to aid a prognosis.

## Figures and Tables

**Table 1:** 3p− phenotypes reported at least twice in DECIPHER patients (15/07/2012).

3p− phenotype reported in DECIPHER patients	Phenotype reported in proband	Also reported in 3q+ syndrome
Microcephaly	− (Mild micrognathia)	−
Ptosis of the eyelids	−	−
MR/DevDel	?	+
Small/short nose	−	−
Seizures	+	+
AVSD	+	+
Clinodactyly/polydactyly	+ (Clinodactyly)	+
Dysmorphic	+	+
Small hands/feet	−	−
Hypotonia	−	−
Hypertelorism	+	−
Feeding problems	+	+
Horseshoe kidney	−	−
Short stature	−	−

DECIPHER patient numbers used: 256371, 249344, 261155, 256542, 251667, 1876, 253652, 249965 253231, 248772 258577, 248715, 248716, 253820, 251867, 253894, 1372, 1213. These data were taken from the DECIPHER Consortium database (http://decipher.sanger.ac.uk/).

## References

[1] Miller DT, Adam MP, Aradhya S (2010). Consensus statement: chromosomal microarray is a first-tier clinical diagnostic test for individuals with developmental disabilities or congenital anomalies. *American Journal of Human Genetics*.

[2] Schinzel A (2001). *Catalogue of Unbalanced Chromosome Aberrations in Man*.

[3] Aqua MS, Rizzu P, Lindsay EA (1995). Duplication 3q syndrome: molecular delineation of the critical region. *American Journal of Medical Genetics*.

[4] Schwyzer U, Binkert F, Caflisch U, Baumgartner B, Schinzel A (1987). Terminal deletion of the short arm of chromosome 3, del(3pter-p25): a recognizable syndrome. *Helvetica Paediatrica Acta*.

[5] Drumheller T, McGillivray BC, Behrner D (1996). Precise localisation of 3p25 breakpoints in four patients with the 3p− syndrome. *Journal of Medical Genetics*.

[6] Green EK, Priestley MD, Waters J, Maliszewska C, Latif F, Maher ER (2000). Detailed mapping of a congenital heart disease gene in chromosome 3p25. *Journal of Medical Genetics*.

[7] Van Essen AJ, Kok K, Van Den Berg A (1991). Partial 3q duplication syndrome and assignment of D3S5 to 3q25-3q28. *Human Genetics*.

[8] Frints SGM, Marynen P, Hartmann D (2003). CALL interrupted in a patient with non-specific mental retardation: gene dosage-dependent alteration of murine brain development and behavior. *Human Molecular Genetics*.

[9] Shuib S, McMullan D, Rattenberry E (2009). Microarray based analysis of 3p25-p26 deletions (3p− syndrome). *American Journal of Medical Genetics, Part A*.

[10] Cuoco C, Ronchetto P, Gimelli S (2011). Microarray based analysis of an inherited terminal 3p26.3 deletion, containing only the CHL1 gene, from a normal father to his two affected children. *Orphanet Journal of Rare Diseases*.

[11] Nienhaus H, Mau U, Zang KD (1992). Infant with del(3) (p25-pter): karyotype-phenotype correlation and review of previously reported cases. *American Journal of Medical Genetics*.

[12] Kariya S, Aoji K, Akagi H (2000). A terminal deletion of the short arm of chromosome 3: karyotype 46, XY, del (3) (p25-pter); a case report and literature review. *International Journal of Pediatric Otorhinolaryngology*.

[13] Cargile CB, Goh DLM, Goodman BK (2002). Molecular cytogenetic characterization of a subtle interstitial del(3)(p25.3p26.2) in a patient with deletion 3p syndrome. *American Journal of Medical Genetics*.

[14] Gunnarsson C, Foyn Bruun C (2010). Molecular characterization and clinical features of a patient with an interstitial deletion of 3p25.3-p26.1. *American Journal of Medical Genetics, Part A*.

[15] Narahara K, Kikkawa K, Murakami M (1990). Loss of the 3p25.3 band is critical in the manifestation of del(3p) syndrome: karyotype-phenotype correlation in cases with deficiency of the distal portion of the short arm of chromosome 3. *American Journal of Medical Genetics*.

[16] Malmgren H, Sahlén S, Wide K, Lundvall M, Blennow E (2007). Distal 3p deletion syndrome: detailed molecular cytogenetic and clinical characterization of three small distal deletions and review. *American Journal of Medical Genetics Part A*.

[17] McCullough BJ, Adams JC, Shilling DJ, Feeney MP, Sie KCY, Tempel BL (2007). 3p− syndrome defines a hearing loss locus in 3p25.3. *Hearing Research*.

[18] Carayol J, Sacco R, Tores F (2011). Converging evidence for an association of ATP2B2 allelic variants with autism in male subjects. *Biological Psychiatry*.

[19] Fernandez T, Morgan T, Davis N (2004). Disruption of contactin 4 (CNTN4) results in developmental delay and other features of 3p deletion syndrome. *American Journal of Human Genetics*.

